# Application of New Sensor Technology and Few-Shot Learning in Education Based on IoT Era

**DOI:** 10.1155/2022/3965416

**Published:** 2022-07-04

**Authors:** Jianwen Feng

**Affiliations:** Dean's Office, Hanshan Normal University, Chaozhou 521041, Guangdong, China

## Abstract

In recent years, with the rapid development of emerging Internet of Things technology and short-range wireless communication technology, smart healthcare monitoring network technology has become a research hotspot. It provides convenience for people and enhances the development of people's own healthcare awareness. This paper aims to study how to make its application in the field of smart healthcare education more applicable through the use of related technologies in the Internet of Things era and few-shot learning. For this reason, this paper proposes to optimize and improve the new sensor technology and the algorithm of few-shot learning, and to adjust some parameters as a whole. At the same time, related experiments and analysis are designed for the improved algorithm to study and understand its performance. The experimental results in this paper show that the improved algorithm improves its application effect by 36.9% and is relatively more applicable than the unimproved algorithm.

## 1. Introduction

Intelligent medical care is a new medical service sharing system that combines computer technology, communication network technology, and modern medical technology. Smart healthcare is designed to serve as a link between patients and hospital specialists, allowing patients to seek advice from specialists in distant hospitals at any time and from any location, and to receive effective treatment and healthcare, as well as other medical services, based on recommendations. It achieves “zero distance” consultation between specific medical costs and time, and patients and hospital experts and medical personnel, and improves medical resource efficiency and distribution rationality.

Intelligent medical monitoring can continuously send human physiological parameter information to a medical monitoring center in a remote hospital for analysis and diagnosis by experts or medical personnel using medical sensors and communication networks. As a result, hospital experts and medical personnel can quickly obtain the patient's testing results, record historical data, and describe reasonable and correct diagnoses and treatment options. In the hospital's database center, the function of long-term tracking of the patient's physical condition can also be realized. The demand for remote medical monitoring is increasing, and the market prospect is broad, thanks to the rise of network applications of medical supplies and the gradual improvement of people's own health awareness.

The novelty of this paper is that it adjusts the classification of the algorithm and other related parameters based on research and understanding of new sensor technology in the emerging Internet of Things era, combined with algorithm optimization for few-shot learning. It makes the improved algorithm's application effect in the actual field of education more applicable, and it can play its role more effectively.

## 2. Related Work

With the rapid development of Internet-related technologies, people have a wide range of applications for decorrelation. As one of the main extensions of the Internet of Things technology, sensor technology has never been interrupted. Mokhtari et al. proposed a new body recognition sensor that can effectively distinguish multiple residents in a home environment to detect their height as a unique biometric. The sensor includes three sensing/communication modules: a pyroelectric infrared (PIR) occupancy, an ultrasonic array, and a Bluetooth Low Energy (BLE) communication module. The PIR occupancy module is used to detect the moving direction, while the ultrasonic array module is used to detect the height of the moving residents [1]. Shimada proposes that, with the application of magnetic fields and magnetically responsive fluids such as magnetic composite fluids (MCFs) as fillers, the effect of electrolytic polymerization on NR-latex such as plastic-type polymer solutions is enhanced. The current new MCF rubber vulcanization method is efficient enough to be widely used in tactile sensors in robotics and engineering applications [2]. Miao et al. believe that new sensor technologies such as near-infrared spectroscopy, chemical imaging, electronic nose, and electronic tongue play an important role in the quality evaluation of traditional Chinese medicine and have prospects and opportunities for future research [3]. Ebrahimi and Mardani propose a new, simple, and inexpensive sensor to detect multipoint contact of a typical robotic wheel. The new sensor enables wheeled robots to scan surfaces and find stability margins during real-time motion without the need for cameras or laser sensors. In addition, it enhances the real-time solution capability of dynamic equations [4]. Chaiendoo et al. proposed a selective colorimetric method for the detection of formaldehyde (FA) based on polymethacrylic acid- (PMAA-) templated silver nanoclusters (AgNCs). In the presence of AgNCs (AgNCs@Tollens), chemical dosimeters are easily fabricated by forming Tollens reagents. Compared with other aldehyde-containing compounds, the proposed method exhibits excellent selectivity for FA [5]. Kuchlyan et al. proposed that Mercury ions pose a great threat to humans due to their high toxicity in living systems. Therefore, its detection at the nanometer level is of current interest. Rhodamine derivatives are one of the rarest examples of Hg2+ fluorescent chemosensors, in which the phthalic acid moiety showing antibacterial activity is responsible for specific binding [6]. Wu and Yan believe that adaptive immune cells are usually not equipped with pattern recognition receptors. In immunity, they revealed an “innate-like” cytosolic DNA sensing mechanism of KU complexes in senescent CD4+ T cells, which exacerbates senescence-related autoimmunity [7]. The new sensor-based wearable technology proposed by Monje et al. is gradually revolutionizing PD care by objectively measuring these manifestations and improving PD diagnosis and treatment monitoring. However, their use in clinical practice remains limited, probably due to the lack of external validation and standards for their continued use at home [8]. The abovementioned articles are very comprehensive for the introduction of related new sensors, and the structure of the sensor and its specific design principles are clearly expressed. However, there is no experimental verification for the application of the sensor in related fields, and there is a lack of research on the reliability of the sensor.

## 3. Application Method of Few-Shot Learning in Smart Health

### 3.1. Smart Healthcare System

The smart healthcare system [9] is to monitor the basic parameters of the human body's important physiological parameters such as human body temperature, pulse, and blood pressure. These parameters can reflect a basic health condition of the human body, so the system chooses to monitor the parameters of the human body's body temperature, pulse, blood pressure, and blood oxygen. The most important part of the system is to transmit the collected data. Data transmission is generally divided into two forms: wired transmission and wireless transmission. The cost of wired transmission is high, the wiring is cumbersome, and the scalability is not very good. When new equipment is added, rewiring may be required, and the disturbed lines may cause psychological pressure on the ward [10]. The wireless transmission method is relatively simple in terms of placement, because it does not require cables, so the cost is relatively low, and the adaptability and scalability are good. It does not require rewiring, and because there are no complicated wires, the instrument is easy to carry and move. This system is designed to be able to monitor their own health at home, requiring the instrument to be portable, simple to operate, simple and easy to install, and easy to expand [11]. According to the above comparison of wired transmission and wireless transmission, the wireless transmission mode is selected. It replaces the cables used in the traditional monitoring system, which not only facilitates the movement of the ward but also reduces the trouble and psychological pressure caused by the disturbed wires.

**Figure 1:**
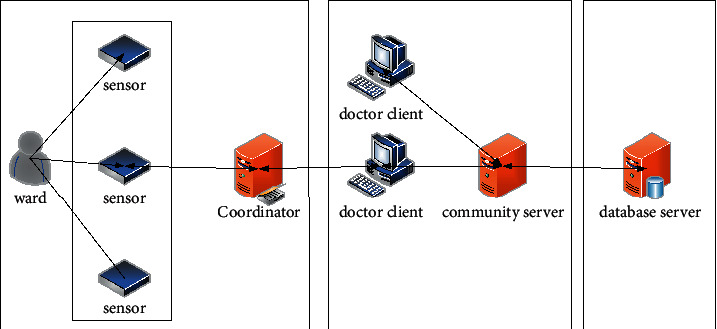
System structure block diagram.

The system is mainly composed of three parts: parameter acquisition and transmission system, client monitoring software system, database management system. The parameter acquisition and transmission system is composed of parameter acquisition sensors, Zigbee acquisition nodes, and coordinators, which are responsible for acquiring the health parameters of the monitored person, such as pulse, blood pressure, body temperature, and other parameter data [12]. Each acquisition node transmits the collected data to the coordinator through the wireless network, and the coordinator processes the data and sends it to the PC serial port through RS232. The client monitoring software system displays and stores the data received by the serial port. At the same time, the client also has the functions of video consultation and health parameter historical data and abnormal data query, which constitute the diagnosis and treatment subsystem. The client also provides doctor data and electronic medical records, which constitute the data query subsystem. The database management system mainly stores and performs simple data processing, deletion, addition, and query of user information and parameter data [13]. The block diagram of the system structure is shown in Figure 1.

#### 3.1.1. Selection of Wireless Transmission Methods

**Figure 2:**
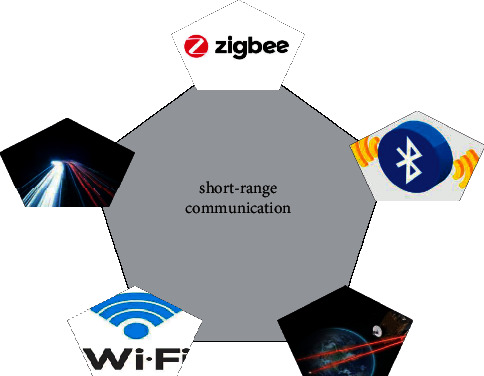
Common centralized short-distance communication methods.

In recent years, with the rapid development of technology and network, especially the rapid development of the Internet industry, the popularity of portable electronic products such as mobile phones and laptop computers has been quite high. People's requirements for network communication are gradually increasing, and wired communication can no longer meet people's mobile needs. They hope to obtain mobile, convenient, and short-distance wireless networks to provide services for these mobile electronic products [14]. The current more common centralized short-range wireless communication technology is shown in Figure 2.

#### 3.1.2. Selection of Network Topology

**Figure 3:**
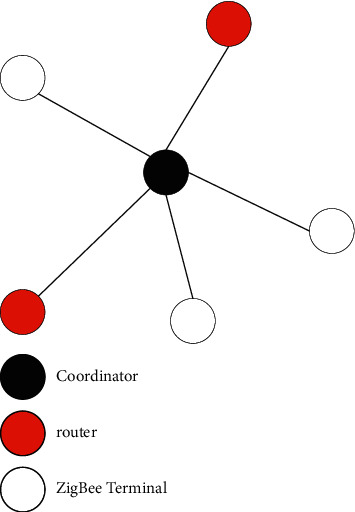
Star network topology.

At present, Zigbee mainly supports star, tree, and mesh network topologies, which constitute three network structures of star network, tree network, and mesh network [15]. The schematic diagram of the star network topology is shown in Figure 3.

**Figure 4:**
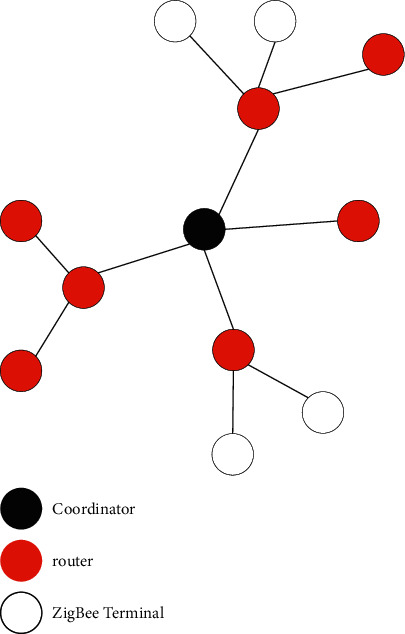
Tree network.

The star network belongs to the slave-master-slave structure, and each wireless network node realizes the network connection through the coordinator. Communication between nodes can only be done through the coordinator. There is no direct communication between nodes, only through the coordinator, which belongs to the point-to-point communication mode [16]. When an FFD is activated, it will automatically form a network and become the main coordinator of the entire Zigbee network. Only one master coordinator is allowed in each Zigbee star network structure, which means that each star network is independent. The uniqueness of the network is ensured by selecting a PAN identifier. After selecting the PAN identifier, the master coordinator will allow the FFD or RFD to join the network. In addition, when multiple wireless nodes transmit data messages to the coordinator at the same time, it is easy to cause network congestion, resulting in data loss or transmission failure. However, it is still widely used due to its advantages of simple structure, flexible layout, and convenient management. The star network structure is suitable for occasions with a small range and few terminal devices [17]. The tree network topology is different. The schematic diagram of the tree network topology is shown in Figure 4.

In a tree-shaped network topology, the communication between nodes can only be carried out along the path of the tree; that is, each node can only communicate with its parent and child nodes. If two nodes have the same parent node, the data will be transmitted from one node to the parent node during data transmission, and then from the parent node to another node [18]. If two nodes have different parents, the information will be passed up the tree path. When it is passed to the nearest ancestor node, it finds the parent node of the target node along the path and then continues to pass down to the final target node.

**Figure 5:**
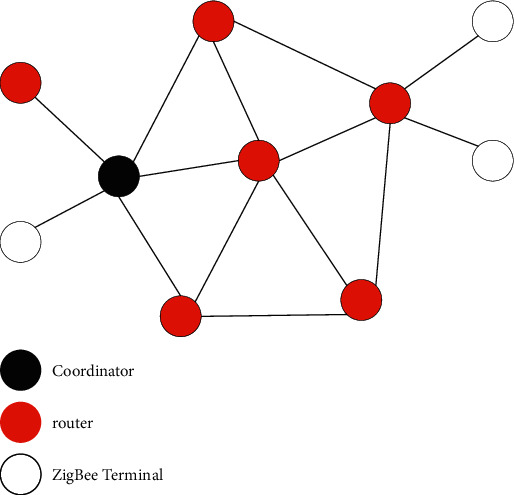
Network structure.

A mesh network is more flexible than a tree network. Similar to the tree network structure, it also includes a coordinator, multiple routers, and multiple terminal nodes, but the connection method is different [19]. The schematic diagram of the mesh network topology is shown in Figure 5.

Most of the nodes in the mesh structure are FFDs with complete functional characteristics. Each FFD node can communicate directly; it can not only send and receive information but also automatically forward the information to other nodes in the network. FFD has the function of rerouting. When a node in the network fails, the nearby nodes will automatically replace the faulty node and continue to transmit information according to the principle of optimal path. The “multihop” mechanism is adopted when the mesh network transmits data, which greatly increases the reliability of the system, increases the security in the process of data transmission, and reduces the delay of data transmission. The mesh network structure is relatively complex, has self-organization and self-healing ability, and can adapt to extremely complex environments [20]. It is suitable for a wide range of applications with complex environments.

### 3.2. Few-Shot Improved Algorithm

Since the introduction of linear discriminant analysis, a lot of supervised learning has been developed in the past few decades to improve the few-shot problem. Representative ones include subspace selection based on geometric mean, local sensitive discriminant analysis, and edge analysis [21]. GMSS uses the general mean definition to replace the arithmetic mean in LDA, so it can get more robust results than LDA. LSDA and MFA are classical manifold learning algorithms, and both have their own solutions to the SSS problem. The PAF framework can better sort out the essential differences of this kind of manifold learning. Although the local geometric information preservation and how to extract discriminative information are fully discussed in current algorithms, the hyperparameter selection problem that exists in most manifold supervised dimensionality reduction is rarely addressed.

Based on the findings, this paper proposes a new essential manifold estimation method based on the RPDA algorithm and ensemble learning that preserves the ensemble manifold's ordering information. This method investigates the problem of supervised learning from two perspectives. To begin, the objective function is optimized on the block based on the RPDA algorithm; that is, the preservation of the ordering information of the same samples is emphasized on the local block formed by the sample, in order to achieve the goal of obtaining the sample's original distribution information, while maximizing sample edge distance between classes in order to retain discriminative information. Second, a new objective function with essential manifold estimation is defined based on the previous step, and the essential manifold of the data is approximated by finding the optimal linear combination of registration matrices, avoiding the hyperparameter selection problem in the traditional manifold supervised learning algorithm.

The ranking information of the samples within the class is available for classification. Inspired by the LE algorithm, we define the intraclass ordering information on a local block as

(1) $$\begin{array}{c} R\left(y_{i}\right)={\sum_{j=1}^{k_{1}}\left\|y_{i}-y_{i^{j}}\right\|}^{2}{\left(w_{i}\right)}_{j}. \end{array}$$

Here the weight factor (*w*_*i*_)_*j*_ is defined as

(2) $$\begin{array}{c} {\left(w_{i}\right)}_{j}=\left\{\begin{array}{cc} \exp\left(\frac{-{\left\|x_{i}-x_{i^{j}}\right\|}^{2}}{t}\right), & \text{if }x_{i^{j}}\in N_{k_{1}}\left(x_{i}\right);\\ 0, & \text{otherwise.} \end{array}\right.. \end{array}$$

According to the PAF framework, we can deduce the formula as follows:

(3) $$\begin{array}{c} R\left(y_{i}\right)=tr\left\{\left[\begin{array}{c} {\left(y_{i}-y_{i^{1}}\right)}^{T}\\ \vdots \\ {\left(y_{i}-y_{i^{j}}\right)}^{T} \end{array}\right]\text{diag}\left(w_{i}\right)\left[y_{i}-y_{i^{1}},\text{…},y_{i}-y_{i^{j}}\right]\right\},\\ =tr\left(Y_{R\left(i\right)}L_{R\left(i\right)}Y_{R\left(i\right)}^{T}\right). \end{array}$$

Here,

(4) $$\begin{array}{c} L_{R\left(i\right)}=\left[\begin{array}{c} -e_{k_{1}}^{T}\\ I_{k_{1}} \end{array}\right]\text{diag}\left(w_{i}\right)\left[\begin{array}{cc} -e_{k_{1}} & I_{k_{1}} \end{array}\right]. \end{array}$$

For popular supervised learning, discriminative information plays a crucial role. As far as the EMRP algorithm is concerned, we consider the purpose of extracting discriminative information by maximizing the distance sum of *y*_*i*_ and its *k*_2_ interclass samples, namely,

(5) $$\begin{array}{c} D\left(y_{i}\right)={\sum_{p=1}^{k_{2}}\left\|y_{i}-y_{i_{p}}\right\|}^{2}{\left(v_{i}\right)}_{j}. \end{array}$$

The definition of the weight factor (*v*_*i*_)_*j*_ here considers the factor of removing the interclass ranking information, namely,

(6) $$\begin{array}{c} {\left(v_{i}\right)}_{j}=\left\{\begin{array}{cc} 1, & \text{if }x_{i^{j}}\in N_{k_{1}}\left(x_{i}\right);\\ 0, & \text{otherwisel.} \end{array}\right. \end{array}$$

Under the framework of PAF, we can further derive (5):

(7) $$\begin{array}{c} D\left(y_{i}\right)=tr\left\{\left[\begin{array}{c} {\left(y_{i}-y_{i_{1}}\right)}^{T}\\ \vdots \\ {\left(y_{i}-y_{i_{p}}\right)}^{T} \end{array}\right]\text{diag}\left(v_{i}\right)\left[y_{i}-y_{i_{1}},\ldots ,y_{i}-y_{i_{p}}\right]\right\},\\ =tr\left(Y_{D\left(i\right)}L_{D\left(i\right)}Y_{D\left(i\right)}^{T}\right). \end{array}$$

Here,

(8) $$\begin{array}{c} L_{D\left(i\right)}=\left[\begin{array}{c} -e_{k_{2}}^{T}\\ I_{k_{2}} \end{array}\right]\text{diag}\left(v_{i}\right)\left[\begin{array}{cc} -e_{k_{2}} & I_{k_{2}} \end{array}\right]. \end{array}$$

Therefore, we can get the optimization objective function on the local block:

(9) $$\begin{array}{c} \arg\underset{y_{i}}{\min}\left(\sum_{j=1}^{k_{1}}{\left\|y_{i}-y_{i^{j}}\right\|}^{2}{\left(w_{i}\right)}_{j}-\gamma \sum_{p=1}^{k_{2}}{\left\|y_{i}-y_{i_{p}}\right\|}^{2}{\left(v_{i}\right)}_{j}\right). \end{array}$$

Here, *γ* ∈ [0,1] is the balance parameter used to synthesize the contributions of intraclass samples and interclass samples on the local patch. By defining an indexed collection,

(10) $$\begin{array}{c} F_{i}=\left\{i,i^{1},i^{2},\text{…},i^{k_{1}},i_{1},i_{2},\text{…},i_{k_{2}}\right\}. \end{array}$$

And we derive (9):

(11) $$\begin{array}{c} \underset{y_{i}}{\arg\min}{\sum_{j=1}^{k_{1}}{\left\|y_{i}-y_{i_{j}}\right\|}^{2}{\left(w_{i}\right)}_{j}-\gamma \sum_{p=1}^{k_{2}}\left\|y_{i}-y_{i_{j}}\right\|}^{2}{\left(v_{i}\right)}_{j},\\ =\underset{y_{i}}{\arg\min tr}\left\{Y_{i}\left[\begin{array}{c} -e_{k_{1}+k_{2}}^{T}\\ I_{k_{1}+k_{2}} \end{array}\right]\text{diag}\left(w_{i}\right)\left[\begin{array}{cc} -e_{k_{1}+k_{2}} & I_{k_{1}+k_{2}} \end{array}\right]Y_{i}^{T}\right\}. \end{array}$$

Here, *tr*( ) is the matrix trace operator:

(12) $$\begin{array}{c} e_{k_{1}+k_{2}}={\left[1,\text{…},1\right]}^{T}\in R^{k_{1}+k_{2}}. \end{array}$$

According to the PAF framework, we further define the registration matrix:

(13) $$\begin{array}{c} L_{i}=\left[\begin{array}{c} -e_{k_{1}+k_{2}}^{T}\\ I_{k_{1}+k_{2}} \end{array}\right]\text{diag}\left(w_{i}\right)\left[\begin{array}{cc} -e_{k_{1}+k_{2}} & I_{k_{1}+k_{2}} \end{array}\right]. \end{array}$$

Formula (11) can be simplified to

(14) $$\begin{array}{c} \underset{Y_{i}}{\arg\min}tr\left(Y_{i}L_{i}Y_{i}^{T}\right). \end{array}$$

We define the selection matrix:

(15) $$\begin{array}{c} {\left(S_{i}\right)}_{pq}=\left\{\begin{array}{cc} 1, & \text{If }p=F_{i}\left\{q\right\};\\ 0, & \text{else.} \end{array}\right. \end{array}$$

Here,

(16) $$\begin{array}{c} \text{where}S_{i}\in R^{N\times \left(k_{1}+k_{2}+1\right)}. \end{array}$$

Subsequently, we can unify all local blocks on a consistent coordinate system. The specific operation method is as follows: first, coordinate *Y*_*i*_ comes from the global coordinate system, and its coordinate expression can be given by the following formula:

(17) $$\begin{array}{c} Y=U^{T}X=\left[y_{1},y_{2},\text{…}y_{N}\right]\in R^{d\times N}. \end{array}$$

That is,

(18) $$\begin{array}{c} Y_{i}=YS_{i}. \end{array}$$

Therefore, the optimization objective function 14 on the local block can be written as

(19) $$\begin{array}{c} \underset{Y}{\arg\min}tr\left(YS_{i}L_{i}S_{i}^{T}Y^{T}\right). \end{array}$$

Secondly, by accumulating the optimization objective function 19 defined on the local block formed by N samples, we can obtain the global registration objective function, which has the following form:

(20) $$\begin{array}{c} \underset{Y}{\arg\min}\sum_{i=1}^{N}tr\left(YS_{i}L_{i}S_{i}^{T}Y^{T}\right),\\ =\underset{Y}{\arg\min}tr\left(YLY^{T}\right). \end{array}$$

However, different hyperparameters correspond to manifolds with different geometric information, which will seriously affect the final recognition accuracy. Therefore, an automatic manifold estimation method is valuable for solving this problem.

### 3.3. Wireless Sensor Networks

**Figure 6:**
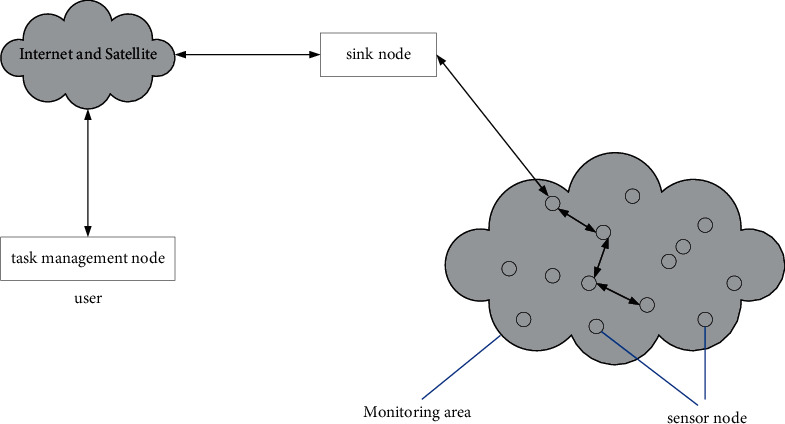
Wireless sensor network architecture.

WSN is a network application system composed of multiple intelligent nodes centrally arranged in a monitoring area, and a multihop self-organized network system formed by wireless communication. It is widely used in facility security, environmental monitoring, industrial applications, traffic control, etc. Therefore, WSN can be said to combine the world of logical information with the world of objective physics, changing the interaction between humans and nature. The wireless sensor network structure is shown in Figure 6.

A sensor network system usually includes sensor nodes, sink nodes, and management nodes. A large number of sensor nodes are randomly deployed in the detection area to form a network in a self-organizing manner. It transmits the detected data to the sink node through multihop relay and finally reaches the management node through the Internet or satellite. The measured data is sent to the sink node and finally reaches the management node through the Internet or satellite. The user configures and manages the sensor network through the management node, publishes detection tasks, and collects detection data.

## 4. Algorithm Module Implementation Experiment

### 4.1. Heart Sound Signal Recognition Experiment

*Production Mechanism*. The vibrations of the heart valves and great vessels under the impact of blood flow pass through the cardiothoracic conduction system to the chest wall to form heart sounds that can be received by a stethoscope. The wavelet transform method is used for denoising, and then the envelope algorithm is used to extract the peaks. It defines the feature vector and then uses the multiclass support vector machine decision tree (SVM-DT) for pattern recognition to distinguish the type of signal; in order to measure the predictive performance of the classifier for unknown samples, it needs to be tested, and the number in the test set is increased to 50, and *p*=2 is also set. The test results are shown in Table 1.

It can be seen from the above table that the algorithm has good prediction ability for unknown samples while maintaining high training accuracy. Through this method, we have established a correct and relatively complete heart sound recognition algorithm, which can reduce the uncertainty of signal recognition and identify the type of signal accurately and effectively.

### 4.2. Breath Sound Signal Recognition Experiment

There are usually two types of breath sounds: normal breath sounds and additional sounds. The breath sound energy of normal and bronchitis patients are mainly concentrated in the inspiratory phase and less in the expiratory phase; the opposite was true in asthmatic patients. Therefore, we need to study the expiratory phase and the inspiratory phase separately, so that the characteristics of the breath sound signal are more obvious.

Input the sample feature vector group of unknown breath sound type after feature extraction to the learned network, and the output is the classification result as shown in Table 2.

### 4.3. Pulse Signal Recognition Experiment

Firstly, the most primitive sampling sequence is used to discretize the pulse signal, and the signal is decomposed twice by the wavelet transform of the discrete sequence; thresholding and secondary reconstruction of the high-frequency coefficients of the signal to obtain the signal after noise removal; normalizing the denoised signal, extracting the signal envelope, and then defining the eigenvectors; finally using the cluster analysis method for pattern recognition.

The pulse data obtained by the final screening were analyzed by using the classical statistical dynamic K-means cluster analysis function. When *K* = 3, the class centers of the cluster analysis results are shown in Table 3.

Passing N groups of test samples through the test of the training network, the error we get has reached the expected result. This algorithm has a concise idea and fast clustering speed. However, there are still some problems, such as the inappropriate selection of initial agglomeration points, which has a great influence on the clustering results and still needs to be improved.

### 4.4. Fusion Algorithm Experiment

Through the above algorithms, we identify and classify the signals collected by each sensor. In this section, we can obtain the final probability distribution value by calculating each classification result of each sensor and the correlation coefficient in each sensor. *Fusion Idea*. The probability distribution value of each sensor target type obtained by the weighted average method (or template method) is fused through the DS evidence theory, which can reduce the uncertainty of the signal and obtain the user's physical condition more accurately, as shown in Table 4.

After getting the probability distribution values corresponding to the sensors, we need to fuse these probability distribution values. When the intersection proposition is an empty set, the decision result can be obtained through evidence fusion. The fusion process is shown in Table 5.

It can be seen from the above experimental data that the fusion algorithm can significantly reduce the uncertainty and obtain a more accurate physical condition of the patient.

## 5. Multisensor Model Analysis

### 5.1. Model Analysis

This section will quantitatively analyze the difference between mobile agent-based wireless sensor network applications and traditional Client/Server model sensor network applications, focusing on the data transmission model and network energy consumption.

**Figure 7:**
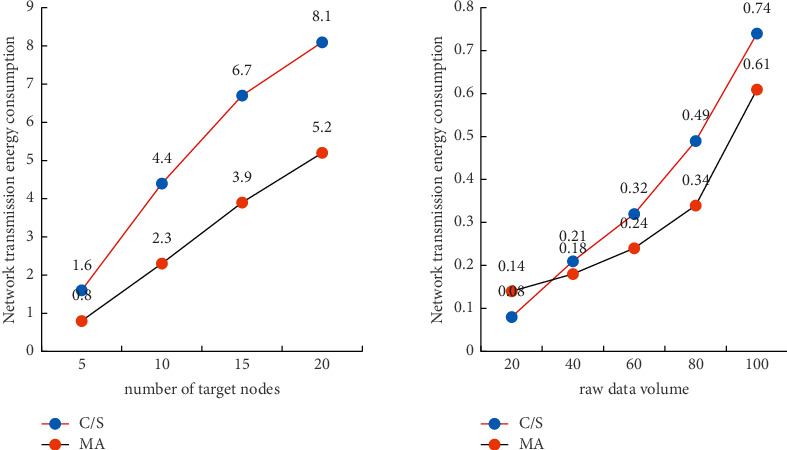
The relationship between the number of target nodes and the amount of raw data, transmission energy consumption, and network energy consumption.

The modules that consume energy in the sensor node include sensor module, processor module and communication module. Since most of the energy consumption of sensor nodes is in the wireless communication module, the energy consumption of the wireless communication module is approximately 30 times the total energy consumption of the sensor module and the processor module. Therefore, the energy consumption of the sensor module and the processor module can be ignored in the energy analysis of the sensor node, and only the energy consumption of the communication module can be considered. In the Client/Server mode and the mobile agent mode, respectively, Figure 7 shows the comparison of the number of target nodes and network energy consumption, as well as the comparison of the original data volume and network energy consumption. The number of target nodes in the network has little effect on the energy consumption of the sensor network using the mobile agent mode, as can be seen on the left side of the figure; however, when the number of target nodes is small, the mobile agent mode may consume more energy. The right side of the figure shows that the mobile agent mode saves more energy than the traditional Client/Server mode as the amount of raw network data grows; however, when the amount of raw data grows small, the mobile proxy mode consumes more energy.

**Figure 8:**
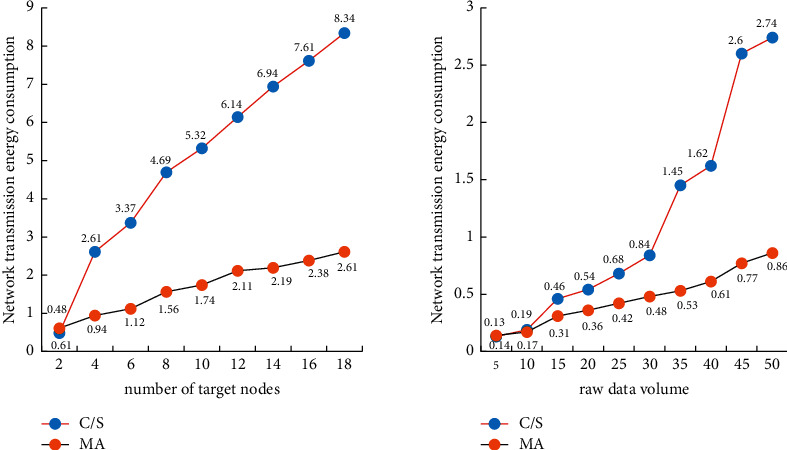
Simulation results between the target node and the original data volume and transmission energy consumption.

At the same time, OMNet is used to simulate the relationship between energy consumption in Client/Server mode and mobile agent mode. Suppose there are 600 nodes irregularly distributed in an area of 300 meters × 300 meters, and the code length of the mobile agent itself is 50 bytes. *kr* is related to the original data volume, the default value is 2 bytes, and the default original data volume of the node is 10 bytes. The specific results are shown in Figure 8: the left side of Figure 8 is the change diagram of network energy consumption during the process of increasing the number of target nodes from 2 to 18.

It can be seen from Figure 8 that the network energy consumption of Client/Server mode and mobile agent mode increases with the increase of the number of target nodes, but the mobile agent mode consumes less energy. Moreover, the growth rate of energy consumption in Client/Server mode is higher than that in mobile agent mode, which is consistent with the above theoretical analysis. The right side of Figure 8 is a simulation diagram of data transmission volume and network energy consumption. The right side of Figure 8 shows that as the amount of raw data increases, the energy consumption of both modes increases. With the increase of the amount of raw data, the energy consumption based on the Client/Server mode increases significantly and exceeds that of the proxy mode, which is also consistent with the above theoretical analysis. When the length of the original data itself is very small compared to the length of the mobile agent code, the Client/Server model has less energy consumption.

### 5.2. Parameter Analysis

**Figure 9:**
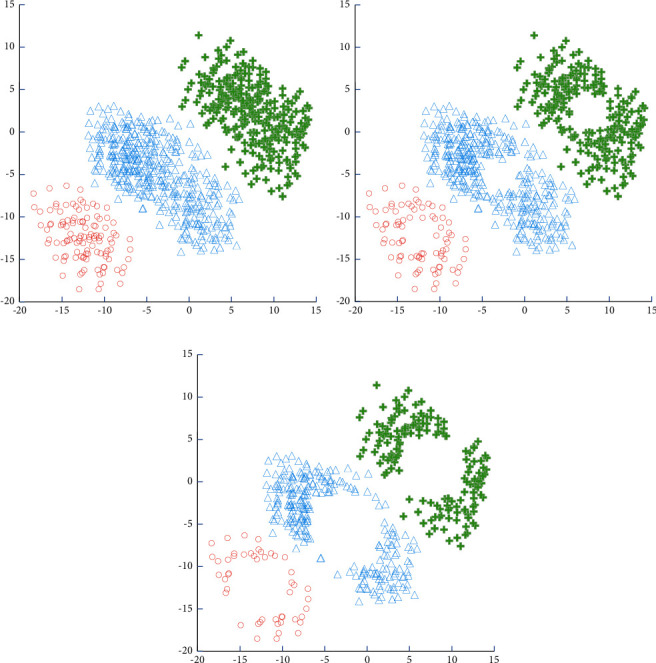
The subset of data selected by the SE algorithm during the iterative process.

In this experiment, a set of two-dimensional datasets is artificially generated, which consists of 1500 instances and contains 3 categories. The dataset shown in Figure 9 shows the instance selection results of the SE algorithm under different reduction rate conditions, which is helpful to observe the SE algorithm process.

In order to further understand the influence of parameters on the performance (accuracy and speed) of SE algorithm, this experiment conducted an in-depth analysis of SE on artificial datasets and Newsgroup datasets.

The SVM classification surface is obviously shifted because a large number of support vectors are accidently deleted. When dealing with multiclassification problems, this type of geometric algorithm must be transformed into multiple binary classification problems for processing: positive classes are considered positive, while negative classes are considered negative. As a result, edge instances in the positive class must be identified with the help of the negative class. It will be very difficult to identify edge instances if the distribution of negative classes surrounds the distribution of positive classes. Furthermore, CNN and KMSVM only retain a small portion of the support vectors, whereas NNSVM only eliminates a few samples that overlap each other.

### 5.3. Application Effect Analysis

**Figure 10:**
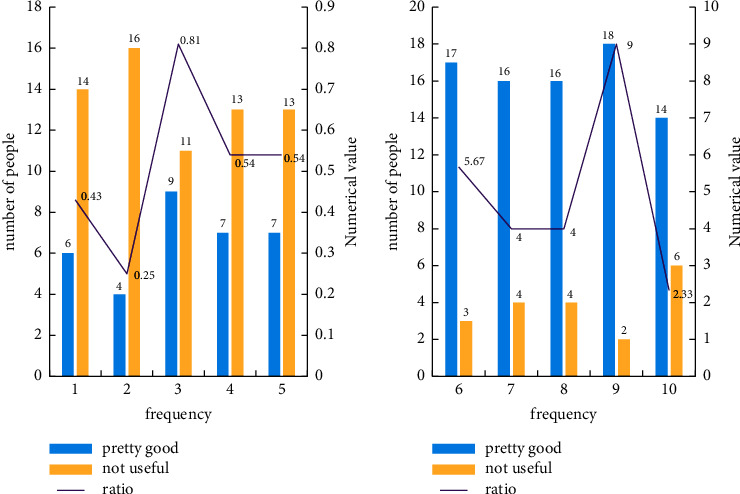
Effect evaluation of few-shot learning algorithm before and after improvement.

In order to better understand the effect of the improved few-shot learning algorithm on the promotion of the new sensor technology in the current Internet of Things era before and after the improvement, this paper designs 10 different experiments, numbered 1–10. The first five experiments are to improve the application effect of the previous algorithm on the new sensor technology on smart healthcare, and the last five experiments are the application effect of the improved algorithm on the new sensor technology on smart healthcare. To ensure that the five experiments are the same except for the sample learning algorithm, other irrelevant variables are the same, and other irrelevant variables are controlled. Statistical experimental data are shown in Figure 10.

From Figure 10 above, we can see that the ratios of good and bad applications considered before the improvement are 0.43, 0.25, 0.81, 0.54, and 0.54, respectively, and the overall ratio is low; after the improvement, the ratios of good and bad applications were considered to be 5.67, 4, 4, 9, and 2.33. The overall ratio is much higher than that before the improvement, and the effect is increased by 36.9%, which can be better applied to the field of smart healthcare education.

## 6. Conclusions

This paper mainly uses the new wireless sensor technology and few-shot learning in the Internet of Things era to explore its application in the field of education. First of all, this paper proposes to improve the algorithm of few-shot learning. By redefining the classification method of the algorithm, the algorithm is optimized. At the same time, it designs relevant experiments to adjust some parameters and conducts data analysis and comparison in the analysis part. From the conclusions of the analysis part, it is not difficult to see that the optimized algorithm has been greatly improved in performance and practical application effect and can be better applied to the application of new sensors in the field of education.

## Figures and Tables

**Table 1:** Calculation results of the classifier during testing.

Heart sound type	Training set		Test set	
	Number of misclassified samples	Accurate %	Number of misclassified samples	Accurate %
*X*	0	100	0	100
*Y*	2	95.2	1	96.7%
*Z*	1	96.4	0	100
Total	3	97.4	1	96.5%

**Table 2:** Classification results.

Type	Number of test samples	Normal	Asthma	Tracheitis	Recognition rate	Average value
Normal	25	23	1	1	92%	92%
Asthma	25	0	22	3	88%	
Tracheitis	25	1	0	24	96%	
Total	75	24	23	28		

**Table 3:** Class centers.

Cluster center	Vascular compliance coefficient	Peripheral resistance coefficient	Aortic valve function coefficient	Cardiac ejection function	Heart rate
First kind	1.61	1.4	0.07	4.6	0.5
Second kind	1.4	1.25	0.05	4.3	0.8
Third kind	1.22	1.01	0.03	3.8	1.2

**Table 4:** Probability assignment values.

	*O *_1_	*O *_2_	*O *_3_	*O *_4_
*m *_1_	0.321	0.1928	0.0913	0.3949
*m *_2_	0.4101	0.234	0.2465	0.1094
*m *_3_	0.51	0.1143	0.0056	0.3701

**Table 5:** Fusion process.

*m*_1_(*u*) = 0.3949	*m*(*o*_1_) = 0.1619	*m*(*o*_2_) 0.0924	*m*(*o*_3_) 0.0973	*m*(*u*) 0.0432
*m*_1_(*o*_3_) 0.0913	*m*(*ϕ*) 0.0374	*m*(*ϕ*) 0.0021	*m*(*o*_3_) 0.0225	*m*(*o*_3_) 0.01
*m*_1_(*o*_2_) 0.1928	*m*(*ϕ*) 0.0791	*m*(*o*_2_) 0.0451	*m*(*ϕ*) 0.0475	*m*(*o*_2_) 0.021
*m*_1_(*o*_1_) 0.321	*m*(*o*_1_) 0.1316	*m*(*ϕ*) 0.0751	*m*(*ϕ*) 0.0791	*m*(*o*_1_) 0.0351
	*m*_2_(*o*_1_) 0.4101	*m*_2_(*o*_1_) 0.2340	*m*_2_(*o*_3_) 0.2465	*m*_2_(*u*) 0.1094

## Data Availability

The data used to support the findings of this study are available from the corresponding author upon request.
